# Amyloid-Beta Radiotracer [^18^F]BF-227 Does Not Bind to Cytoplasmic Glial Inclusions of Postmortem Multiple System Atrophy Brain Tissue

**DOI:** 10.1155/2018/9165458

**Published:** 2018-02-06

**Authors:** Mathieu Verdurand, Elise Levigoureux, Sophie Lancelot, Waël Zeinyeh, Thierry Billard, Isabelle Quadrio, Armand Perret-Liaudet, Luc Zimmer, Fabien Chauveau

**Affiliations:** ^1^Lyon Neuroscience Research Center, CNRS UMR5292, INSERM U1028, Université Claude Bernard Lyon 1, Université de Lyon, Lyon, France; ^2^Hospices Civils de Lyon, Groupement Hospitalier Est, Lyon, France; ^3^Institute of Chemistry and Biochemistry, CNRS UMR5246, Université Claude Bernard Lyon 1, Université de Lyon, Villeurbanne, France; ^4^CERMEP-Imaging Platform, Bron, France

## Abstract

The accumulation of aggregated alpha-synuclein (*α*-syn) in multiple brain regions is a neuropathological hallmark of synucleinopathies. Multiple system atrophy (MSA) is a synucleinopathy characterized by the predominant cerebral accumulation of aggregated *α*-syn as cytoplasmic glial inclusions (CGI). A premortem diagnosis tool would improve early diagnosis and help monitoring disease progression and therapeutic efficacy. One Positron Emission Tomography (PET) study suggested [^11^C]BF-227 as a promising radiotracer for monitoring intracellular *α*-syn deposition in MSA patients. We sought to confirm the binding of this radiotracer to *α*-syn using state-of-the-art autoradiography. Medulla sections were obtained from 9 MSA patients and 9 controls (London Neurodegenerative Diseases Brain Bank). [^18^F]BF-227, chemically identical to [^11^C]BF-227, was used at nanomolar concentrations to perform* in vitro* autoradiography assays. Autoradiograms were superimposed on fluorescent staining from the conformational anti-*α*-syn antibody 5G4 and quantified after immunofluorescence-driven definition of regions of interest. Autoradiography showed no specific signals in MSA patients in comparison to controls despite widespread pathology detected by immunofluorescence. Autoradiography does not support a significant binding of [^18^F]BF-227 to CGI at concentrations typically achieved in PET experiments.

## 1. Introduction

Most neurodegenerative diseases are proteinopathies or protein misfolding diseases that lead to the formation of insoluble protein aggregates in the brain. Proteinopathies with *α*-synuclein (*α*-syn) aggregates form a heterogeneous group of diseases called “synucleinopathies.” Alpha-synuclein is deposited as intracellular inclusions (i) inside neurons in Parkinson's disease (PD), PD with dementia (PDD), and dementia with Lewy bodies (DLB) and (ii) inside oligodendroglial cells in multiple system atrophy (MSA). Subsequent neuropathological lesions are termed Lewy bodies and neurites (LB/LN) and cytoplasmic glial inclusions (CGI), respectively. Alpha-synuclein aggregation is supposed to drive neuronal dysfunction and death and may start long before the first clinical symptoms appear. Therefore,* in vivo* imaging of *α*-syn deposits is a critical need for early and differential diagnosis of synucleinopathies. Quantitative imaging is also a highly awaited tool for the evaluation of disease-modifying drugs targeting *α*-syn [1], as current therapies alleviate symptoms without changing the course of the aggregation process.

During the last ten years, Positron Emission Tomography (PET) has provided continuous advances in detecting the pathognomonic protein aggregates of Alzheimer's disease (AD), namely, the extracellular amyloid-*β* (A*β*) plaques and more recently the intracellular neurofibrillary tangles (NFT). These initial successes have demonstrated the ability of small aromatic radiotracers to detect a new type of target made of large insoluble fibrils of the same endogenous protein. Selectivity has proven to be the main challenge in developing new radiotracers for protein aggregates, as fibrillation in the form of *β*-sheet stacking is a common pathway to protein aggregation.

Several research groups (including ours) have reported aborted attempts in identifying a radioligand binding with sufficient affinity and selectivity for human *α*-syn deposits [2–5]. Thus, despite the implementation of several international consortia, there is no radiotracer that unequivocally detects *α*-syn deposition. To the best of our knowledge, only one study reported PET detection of CGI in MSA patients versus controls (CTL), using the A*β* radioligand [^11^C]BF-227 [6]. Though, no autoradiography data were available to support the PET findings, and to date, these results have not been replicated nor extended to *α*-syn deposition in other synucleinopathies like PD, PDD, or DLB. Another study used an identical ligand with a fluorine-18 labelling, [^18^F]BF-227, and reported conflicting results, that is, fluorescence detection of LB but lack of radioactive binding to DLB homogenates [7].

The present study intended to clarify whether BF-227 binds to *α*-syn CGI in autoradiography experiments using MSA versus CTL brain sections obtained from a single brain bank. MSA was found to be a favourable pathological condition for this evaluation, as midbrain display a high density of CGI (compared to the density of LB/LN in PD/DLB), without A*β* copathology (while this is rather common in PD or DLB).

## 2. Methods

### 2.1. Radiolabelling

Fluorine-18 was obtained via the ^18^O (p,n)^18^F reaction (IBA Cyclone18/9 cyclotron). The fluorosubstitution of tosylate was realized on a standard Neptis® synthesizer (ORA™): after initial fluoride preparation (collection, drying, and Kryptofix activation), 1.0–2.0 mg of BOTs227 radiolabelling precursor was introduced and the reaction mixture was heated at 150°C for 10 min in DMSO. After dilution with 15 mL of water, the reaction mixture was passed through an activated C18 cartridge for prepurification and the crude product was eluted from the cartridge with 1.5 mL of methanol. Pure [^18^F]BF-227 was obtained after separation on a preparative high-performance liquid HPLC (C18 Symmetry Prep Waters 7 *μ*m 7.8 × 300 mm, Waters) eluted with H_3_PO_4_ 20 mM/THF/TFA 80/20/0.1% at 3 mL·min^−1^ (*λ* = 254 nm), with a retention time of 43 min. For biological use, the product was formulated via SPE techniques 51. The dilution of the product was done with 20 mL of sterile water, loaded onto a C18 cartridge (SEP-Pak Light, Waters). The loaded cartridge was rinsed with water, eluted with 1 mL of ethanol, and diluted with isotonic saline to an ethanol concentration of 5%. Quality control consisted in determination of radiochemical purity and specific activity, by analytical HPLC assay (UV and radioactive detections; MachereyeNagel EC 250/4.6 Nucleodur 100-5-C18ec C18 column; elution with H2O/CH3CN/TFA 55/45/0.1% at 0.9 ml·min^−1^ with a retention time of 7.0 min). The identity of [^18^F]BF-227 was confirmed by coinjection with an authentic nonradioactive sample. Specific activities ranged from 40 to 840 GBq/*μ*mol at autoradiography start.

### 2.2. Human Postmortem Brains

Human brain tissue sections were obtained as 20 *μ*m thick frozen sections from the London Neurodegenerative Diseases Brain Bank [8]. Five consecutive medulla sections from nine patients with MSA and nine controls were used (Table 1). Sections from one AD patient (67 y.o. male, from local brain bank in Lyon: Cardiobiotec, CRB-HCL), at the level of cortex and hippocampus, were also included as a positive control for extracellular amyloid deposits.

### 2.3. *In Vitro* Autoradiography

**Figure 1:**
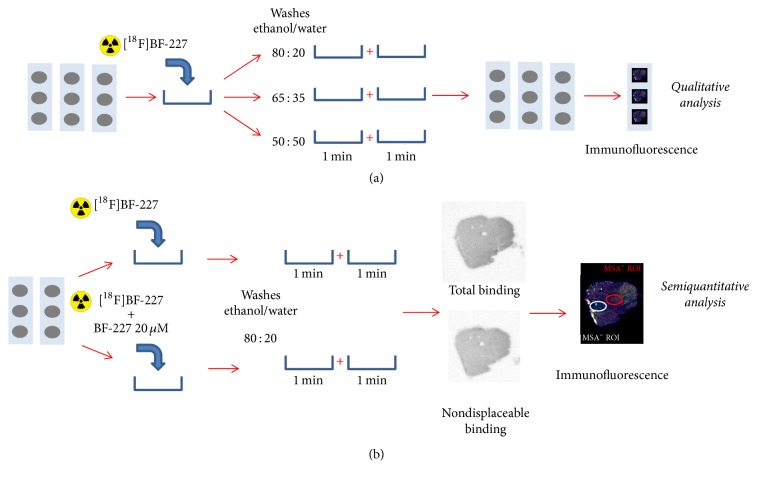
Two-step methodology for autoradiography assays on MSA patients and CTL subjects. Qualitative experiments to optimize washing conditions were first performed (a), followed by quantitative evaluation of total and nondisplaceable binding of [^18^F]BF-227 (b).

A first set of experiments was performed to select the optimal washing conditions minimizing the nonspecific binding (Figure 1(a)). Three consecutive sections per patient were incubated at room temperature (RT) for 30 minutes in phosphate-buffered saline (PBS, Sigma-Aldrich P4417) at pH 7.4 with 27–90 MBq/L (0.1–1.1 nM) of [^18^F]BF-227. After incubation, the three slides were subjected to three different washing conditions: ethanol/water 80 : 20 or 65 : 35 or 50 : 50 (two 1-minute washes at RT). Slides were then dried under cool air and and exposed to sensitive phosphor imaging plate (BAS-IP MS 2025, Fujifilm) overnight. The distribution of radioactivity was then digitised with a bio-imaging analyser system (BAS-5000, Fujifilm).

A second set of experiments was performed with the selected washing conditions (ethanol/water 80 : 20) so as to quantify total and nondisplaceable binding (Figure 1(b)). Two consecutive sections per patient were used: one incubated with [^18^F]BF-227 and the other incubated with [^18^F]BF-227 and 20 *μ*M of unlabeled BF227.

### 2.4. Immunofluorescence

Immunofluorescence was performed on the same sections used for* in vitro* autoradiography. Brain sections were postfixed in 4% paraformaldehyde in PBS (phosphate buffer saline) for 20 minutes followed by 3 washes (5 min each) in PBS and transfer to a PBS solution (0.02% azide) at 4°C until use. Antigen retrieval was conducted with 99% formic acid solution during 10 min followed by 3 washes (5 min each) in PBS. Slides were then dipped in blocking and permeabilization buffer (PBST with 5% BSA and 0.5% Triton X-100) for 30 min. Incubation in PBST containing the primary antibody 5G4 (1/1000; anti-aggregated *α*-synuclein, monoclonal mouse IgG1*κ*, kindly provided by Dr Ingolf Lachmann) was then performed overnight at 4°C. After 3 washes (5 min each) in PBS, slides were then incubated in PBST containing the secondary antibody Alexa Fluor 594 (1/1000; AF594 anti-mouse, LifeTechnologies) for 1 hour at RT followed by 3 washes (5 min each) in PBS. Slides were prepared for observation in an aqueous medium containing DAPI (Roti-Mount FluorCare DAPI, Carl Roth). Fluorescence was captured with a digital slide scanner (Axio Scan.Z1, Zeiss) (CIQLE Imaging Platform, University of Lyon, France).

### 2.5. Quantification and Statistics

Demographic features of MSA patients and CTL subjects were compared with nonparametric Mann–Whitney tests. Autoradiography and fluorescence images were coregistered using the eC-CLEM plugin [9] from Icy [10]. The first set of experiments (different washing conditions) was analysed qualitatively by visual inspection of overlaid images. The second set of experiments (total and nondisplaceable binding) was analysed quantitatively as follows. On MSA sections, two types of regions of interest (ROI), with (MSA^+^) and without (MSA^−^) fluorescent cytoplasmic glial inclusions, were manually defined and reported on autoradiography (1 to 3 ROI per section). On CTL sections, one type of regions of interest (CTL) was drawn similarly. Semiquantitative binding values, in psl/mm^2^ (photostimulated luminescence per surface unit), were extracted and averaged across each section, after background subtraction. Displaceable binding in each ROI was calculated as total binding minus nondisplaceable binding and reported as mean ± standard deviation. The three groups (MSA^+^, MSA^−^, and CTL) were compared with a one-way ANOVA followed by Bonferroni's post hoc tests.

## 3. Results

**Figure 2:**
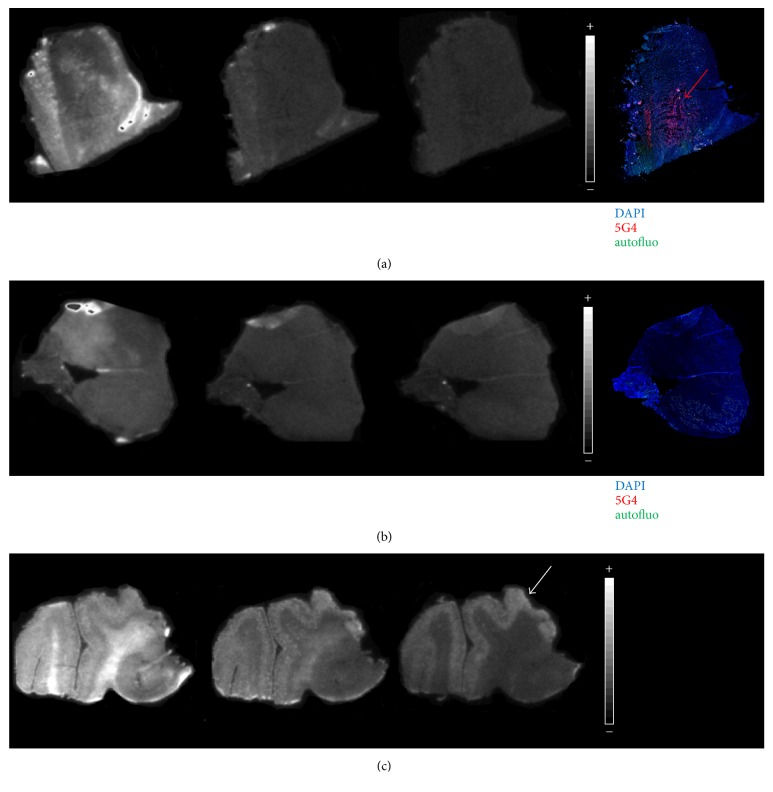
Representative autoradiography images obtained from (a) an MSA patient, (b) a CTL subject, and (c) a patient with Alzheimer's disease used as positive control. Three consecutive medulla sections were incubated with [^18^F]BF-227 and washed with various ethanol concentrations (50-65-80% from left to right). Corresponding immunofluorescence against pathological *α*-syn (in red, red arrow) did not match the autoradiography signals (in white) detected after washes with 50 and 65% ethanol (a, b). In contrast, amyloid plaques in the Alzheimer's patient were best detected (white arrow) after washing the slides with 80% ethanol.

Immunofluorescence with the 5G4 conformational antibody allowed the specific detection of aggregated *α*-syn [11]. All MSA sections (except one, which tissue was found to be damaged; this patient was therefore excluded from the study) presented a high density of *α*-syn staining, with typical flame-shaped morphology. However, staining was heterogeneous across the section area with, for example, olivary bodies being relatively spared by *α*-syn pathology (Figure 2(a)). This heterogeneity was used to define ROI with (MSA^+^) and without (MSA^−^) fluorescent cytoplasmic glial inclusions on each MSA section (Figure 1). Control sections showed no staining; only autofluorescence was visible.

A first set of autoradiography assays explored various ethanol concentrations in washing baths to minimize nonspecific binding. As expected, overall autoradiography signals decreased with increasing ethanol concentration, both in patients and controls. Across each section area, strong inhomogeneous signals were visible with 50% ethanol, fading with 65% and 80% ethanol. No visual correlation was noted between autoradiography and immunofluorescence intensities in MSA sections (Figure 2). Similarly, Alzheimer sections showed a prominent white matter signal with 50% ethanol, which persisted with 65% ethanol, while only A*β* signals in the grey matter were specifically detected with 80% ethanol (Figure 2).

**Figure 3:**
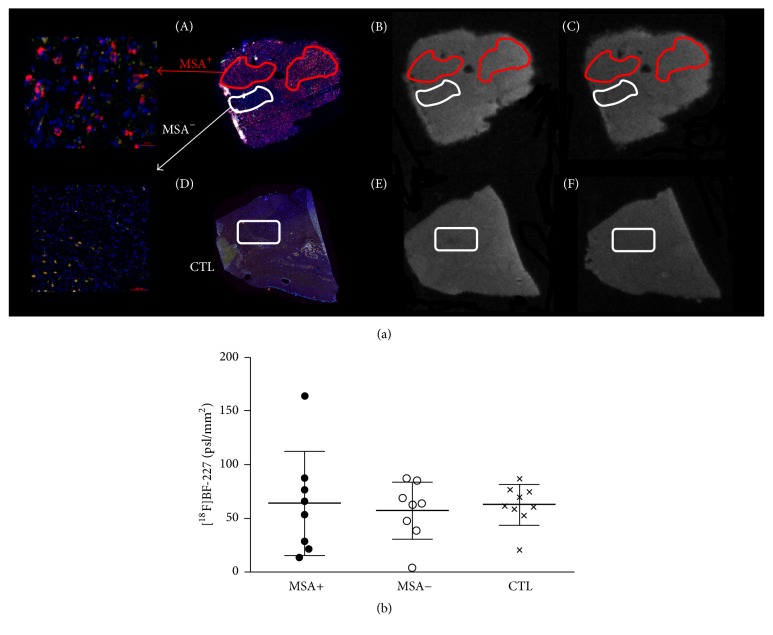
Quantification of total and nondisplaceable [^18^F]BF-227 binding. (a) Regions of interest, with (MSA^+^, in red) and without (MSA^−^, in white) aggregated alpha-synuclein staining were defined according to immunofluorescence ((A) MSA patient and (D) CTL subject). Corresponding total and nondisplaceable binding were extracted from MSA patients (B and C) and CTL subjects (E and F). (b) Computed displaceable [^18^F]BF-227 binding in MSA^+^, MSA^−^, and CTL regions of interest, plotted as individual points (for each MSA patient or CTL subject) and mean ± standard deviation. Differences between the three groups were not significant.

Based on this primary evaluation, a second set of autoradiography assays performed with 80% ethanol in washing baths aimed at quantifying displaceable binding. Mean displaceable binding was very weak (<200 psl/mm^2^) and similar between CTL, and MSA^+^ and MSA^−^ regions of interest (Figure 3).

## 4. Discussion

This study questioned the previously reported PET detection of *α*-syn inclusions in MSA patients using an A*β* radiotracer [6]. This issue is of primary importance for the future development of derivatives showing a higher selectivity for *α*-syn than for A*β*. This work is the first evaluation of [^18^F]BF-227 binding to *α*-syn by autoradiography. MRC London Neurodegenerative Diseases Brain Bank provided fresh-frozen sections of medulla oblongata from MSA patients along with controls without *α*-syn pathology. Medulla oblongata is systematically affected during the course of MSA progression and is, importantly, devoid of A*β* copathology [12].

Radiotracers targeting protein aggregates like A*β* or Tau display a high level of nonspecific binding. Ethanol is commonly used in autoradiography protocols to optimize aggregate detection by increasing the target-to-noise ratio (specific to nonspecific binding) [13, 14]. Here, whatever the ethanol concentration used, autoradiography signals appeared unrelated to CGI density in MSA and similar to controls (Figures 2(a)–2(b)). In contrast, [^18^F]BF227 binding to amyloid plaques was perfectly detectable despite washing with 80% ethanol (Figure 2(c)). Therefore the inability to retain specific binding upon optimized washing conditions suggests that BF-227 does not bind to CGI with nanomolar affinity. Would lower-affinity binding sites to CGI exist and be eliminated with increasing ethanol concentrations, these binding sites are unlikely to contribute to in vivo PET signals. In addition, blocking experiments yielded a very weak displaceable binding, which did not vary with the level of aggregated *α*-syn. Therefore, autoradiography does not support a significant binding of [^18^F]BF-227 to CGI at nanomolar concentrations. This negative result is in line with our previously reported lack of binding in *α*-syn transgenic mice by [^18^F]BF-227 PET [15].

In contrast, two independent studies reported the detection of MSA [6] or DLB [7] *α*-syn inclusions through BF-227 fluorescence. However, this apparent contradiction may not be surprising given that fluorescence requires a micromolar concentration while autoradiography proceeds in the low nanomolar range, a concentration relevant to* in vivo* PET studies. The same discrepancy between fluorescence and autoradiography towards *α*-syn binding has been recently highlighted with a Tau radiotracer [16]. We conducted a similar fluorescent detection in one MSA patient to highlight fluorescent detection of unlabeled BF227 at 100 *μ*M without corresponding autoradiography detection at 1 nM (supplementary material and figure (available here)).

It must be noted that the present study does not disprove the initial PET results in MSA patients but rather indicates that PET differences are unlikely to be related to *α*-syn binding. Additionally, no [^11^C]BF-227 PET binding differences were observed between MSA patients and CTL subjects in the pons (containing medulla oblongata) [6]. Widespread inflammation in MSA patients, also evidenced by PET [17], may be a possible factor influencing [^18^F]BF-227 nonspecific uptake. This difficulty in comparing in vitro and in vivo findings is actually common with radiotracers of protein aggregates (amyloid, Tau, and synuclein), which usually display multiple binding components with differential affinities, along with a high level of nonsaturable binding [18, 19]. Importantly, the present results strengthen the selectivity of BF-227 towards dense-cored A*β*: this is of importance for its use in dementia as mixed A*β*/*α*-syn pathologies frequently coexist in AD as well as in PD or DLB [20].

## 5. Conclusion

We performed gold-standard* in vitro* autoradiography experiments in postmortem brain tissue of confirmed MSA cases. The present data imply that *α*-syn pathology in MSA is unlikely to be detectable by [^18^F]BF-227 PET.

## Figures and Tables

**Table 1:** Demographic features of subjects (*p* value from Mann–Whitney test).

	MSA	CTL	*p value*	AD
Number of cases	9	9	-	1
Gender (M/F)	5/4	5/4	-	M
Age (years)	67.6 ± 2.4	75.1 ± 2.9	0.08	67
Postmortem interval (h)	32.9 ± 7.0	40.1 ± 5.4	0.25	36.25

## Supplementary Materials

Supplementary Materials Figure S1: (A) only tissue autofluorescence (in green) is detected prior to BF-227 incubation; (B) BF-227 fluorescently labelled (in blue) few inclusions which were clearly visualized after washing with 50% ethanol buffer. Washes with PBS without ethanol resulted in whole tissue staining and high background signals (not shown).
