# Supplementary material for: Amyloid-Beta Radiotracer [18F]BF-227 Does Not Bind to Cytoplasmic Glial Inclusions of Postmortem Multiple System Atrophy Brain Tissue
Source: Contrast Media Mol Imaging. 2018 Feb 6;2018:9165458. doi: 10.1155/2018/9165458 (PMC5818909; doi:10.1155/2018/9165458)
Supplement: Supplementary Materials — Figure S1: (A) only tissue autofluorescence (in green) is detected prior to BF-227 incubation; (B) BF-227 fluorescently labelled (in blue) few inclusions which were clearly visualized after washing with 50% ethanol buffer. Washes with PBS without ethanol resulted in whole tissue staining and high background signals (not shown). [file 9165458.f1.pdf]

Supplementary material and figure

Fluorescent detection of BF-227 was performed according to Kikuchi and coll. [6]. Briefly, 1 mg BF-227 was dissolved in 1 ml ethanol and further diluted in PBS to a 100 µM working solution. One fresh-frozen section from a MSA patient (same as Figure 3a) was successively incubated in PBS (5 min), BF-227 solution (30 min), PBS (2 min), and ethanol 50% (2 min). Microscopic examination was performed after each step using an epifluorescence microscope (Axioplan-2, Zeiss) with DAPI (blue, exposure 500 ms) and FITC (green, exposure 100 ms) filters.

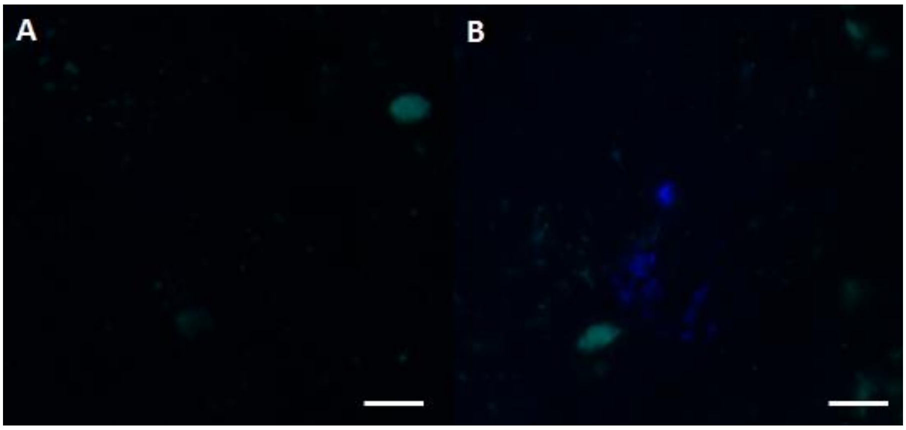

Figure S1. (**A**) Only tissue autofluorescence (in green) is detected prior to BF-227 incubation. (**B**) BF-227 fluorescently labelled (in blue) few inclusions which were clearly visualized after washing with 50% ethanol buffer. Washes with PBS without ethanol resulted in whole tissue staining and high background signals (not shown).
